# The impact of COVID-19 on women’s empowerment: A global perspective

**DOI:** 10.7189/jogh.13.06021

**Published:** 2023-06-16

**Authors:** Juan Dempere, Rihab Grassa

**Affiliations:** 1 Higher Colleges of Technology, Ras Al Khaimah, United Arab Emirates; 2 Higher Colleges of Technology, Dubai, United Arab Emirates

## Abstract

**Background:**

This research explores the repercussions of the COVID-19 emergency on women’s enablement worldwide, scrutinizing data spanning 93 nations between 2019 and 2020.

**Methods:**

The investigation employs sectional data examination of various metrics pertinent to women’s empowerment, encompassing the ratio of employed females to the total populace, women’s labour force involvement, female representation in legislative assemblies, young women disengaged from education, occupation, or skill acquisition, and unemployment rates among women.

**Results:**

The research identifies encouraging and disheartening developments in female empowerment amidst the pandemic. On an optimistic note, there is a growing inclination towards women’s presence on corporate boards, executive and managerial roles, and positions within publicly owned enterprises. Conversely, there is a notable reduction in the ratio of working women to the overall population, a marginal decrease in female labour force engagement, an upswing in young women uninvolved in education, occupation, or skill development, and an elevation in female unemployment rates.

**Conclusions:**

The study’s results accentuate the demand for tailored initiatives and strategies that address the pandemic’s distinct ramifications on women, including backing for female employment, education, and political involvement. The research further emphasizes the significance of sustained endeavours to foster gender diversity within the business arena, where the COVID-19 upheaval has less impeded female empowerment. Legislators, global entities, and community organizations must prioritize and allocate resources toward gender-sensitive policies and actions to alleviate the detrimental impacts of crises on women, advancing their empowerment, adaptability, and engagement across all life spheres.

The COVID-19 pandemic has affected millions worldwide, causing unprecedented health, social, and economic upheavals. The crisis has exposed and exacerbated existing societal inequalities, with women being disproportionately affected by the pandemic’s consequences. Women’s empowerment, a critical element for achieving gender equality and sustainable development, has been severely challenged during the outbreak. The effects of the pandemic on women’s empowerment have been studied in various ways, but they have yet to look at the issue from a global perspective. To fill this gap, this study aims to examine the impact of the COVID-19 crisis on women’s empowerment from a global perspective, analysing data from 93 countries between 2019 and 2020. This research is significant as it is the first of its kind to provide a comprehensive analysis of the effects of the pandemic on multiple dimensions of women’s empowerment, including employment, education, and political representation.

Women’s empowerment is a multifaceted concept encompassing social, economic, and political dimensions. It refers to how women gain access to resources, opportunities, and decision-making power, leading to greater autonomy, self-confidence, and improved quality of life. Women comprise half the global population, so their empowerment is crucial for achieving gender equality and sustainable development. The United Nations [1] has recognized gender equality and women’s empowerment as one of the 17 Sustainable Development Goals (SDGs) under Goal 5: Achieve gender equality and empower all women and girls.

The COVID-19 pandemic has disproportionately affected women in various ways. For instance, women have experienced a more significant loss of employment and income than men, as they are often concentrated in the informal sector and low-paid, insecure jobs. The closure of schools and daycare centres has increased the burden of unpaid care work for women, further exacerbating the gender gap in the labour force. Additionally, the crisis has increased gender-based violence, with reports of domestic violence surging globally during lockdown periods.

In this study, we analyse the impact of the COVID-19 crisis on women’s empowerment by examining various indicators, such as female employment-to-population ratios, female labour force participation, women’s parliamentary representation, female youth unengaged in education, employment, or training, and female unemployment rates. We use a cross-sectional data analysis from 93 countries worldwide between 2019 and 2020 to investigate these relationships. Our findings reveal positive and negative trends in women’s empowerment during the pandemic.

On the positive side, we find an increasing trend in women’s representation on corporate boards, executive and managerial positions, and employment in publicly traded companies. This development suggests that the COVID-19 crisis has not significantly impacted corporate women’s empowerment, and efforts to promote gender diversity in the corporate sector have continued during the pandemic.

However, our study also reveals several negative consequences of the COVID-19 crisis on women’s empowerment at the community level. We find a significant decrease in female employment-to-population ratios and a slight decline in female labour force participation. Moreover, the share of female youth not engaged in education, employment, or training has increased, indicating that the pandemic has adversely affected women’s access to education and employment opportunities. Furthermore, our results show a rise in female unemployment rates and an increase in women’s parliamentary representation, suggesting a complex relationship between the crisis and women’s political empowerment.

These findings have significant implications for policymakers and stakeholders promoting gender equality and women’s empowerment. Our results highlight the urgent need for targeted interventions and policies that address the pandemic’s specific effects on women, including support for women’s employment, education, and political participation. Additionally, our study underscores the importance of continued efforts to promote gender diversity in the corporate sector, as women’s empowerment in this domain has been less affected by the COVID-19 crisis.

In summary, our research provides a comprehensive analysis of the impact of the COVID-19 pandemic on women’s empowerment from a global perspective, contributing valuable insights to inform policy and decision-making in the ongoing efforts to address gender inequalities. By identifying the positive and negative consequences of the pandemic on women's empowerment, our study offers a nuanced understanding of women's challenges and opportunities during this unprecedented global crisis. As the world grapples with the effects of COVID-19 and prepares for future pandemics, governments, international organizations, and civil society must prioritize and invest in gender-responsive policies and interventions. These efforts should mitigate the adverse effects of crises on women and promote their empowerment, resilience, and participation in all aspects of life. By doing so, we can work towards building a more inclusive, equitable, and sustainable post-pandemic world that leaves no one behind.

The growing literature on COVID-19’s economic effects highlights severe financial losses for businesses globally. Fairlie [2] reported a 22% decline in USA small business activity from February to April 2020, disproportionately affecting minority groups. Fairlie and Foseen [3] noted that lockdowns severely affected some California industries while others, like online sales, prospered. Dempere [4] observed that countries with greater business freedom had laxer restrictions and higher cases and deaths.

From a labour perspective, the outbreak resulted in significant job losses globally. Dempere [4] informs of a meaningful association between a country’s labour freedom and its daily mean of COVID-19 infections per million. He proposes that governments with squat labour freedom endured elevated unemployment demanding state interference and controls. Bell and Blanchflower [5] report that about 33% of American and Canadian labourers lost around 50% of their pay because of the COVID-19 crisis, contrasted with 25% in the UK and 45% in China. Su et al. [6] report a strong link between COVID-19 and joblessness in Italy, Germany, and the UK, while Mekonnen and Kassegn [7] note a 2020 working hour loss of over 10% in Eritrea, Cape Verde, and Uganda. To protect existing jobs, governments implemented work retention agreements and suspensions on firings [8].

The COVID-19 crisis has necessitated stringent economic measures in many countries. Dempere [4] found a substantial negative correlation between a nation’s monetary freedom and its pandemic response time, yet a positive correlation between monetary freedom and the daily mean of COVID-19 infections per million. He suggests that during the initial stages of the pandemic, countries worldwide suffered scarcities and price increases of several goods due to arrested manufacturing, rotting harvests caused by lack of gathering, international supply-chain disruptions, etcetera. The World Health Organization (WHO) [9] invited businesses and governments to align policies to curb amplified product demand due to panic procurements, speculation, and hoarding, especially for health-related supplies. Ahn [10] states that South Korea imposed a fixed retail price on medical masks to prioritize public health and stop the outbreak spread. Chakraborti and Gavin [11] found a positive correlation between USA banning price gouging and shortages of such regulated products. Islam et al. [12] claim that the perceived amount and time scarcity heightened fear-driven consumer buying behaviour in the USA, China, India, and Pakistan. Hall et al. [13] demonstrate stockpiling shopper behaviours during the COVID-19 crisis with increased expenditure in some buyer goods classes.

The outbreak posed challenges to world trade. Dempere [4] notes a correlation between trade freedom and COVID-19 fatalities. Carreño et al. [14] report that some nations were resorting to trade controls. Hoekman et al. [15] document 75 countries, incl. the USA, China, EU, India, Turkey, UK, having imposed trade restrictions on medical materials / drugs. Pauwelyn [16] reports that 69 nations were curtailing personal protective equipment, pharmaceuticals, and health supplies exports.

Politically, the pandemic imposed significant restrictions on civil liberties in many countries. Dempere and Modugu [17] found a positive, significant correlation between daily COVID cases and fatalities per million and a nation’s communication technology readiness. This finding supports the idea that false information during the early pandemic caused substantial problems for government control strategies. Dempere [18] recommends that non-political institutions participate in effective crisis management systems. In addition, Dempere [19] finds that democracies implemented the least stringent social restrictions during the first wave of COVID-19, as measured by the daily stringency index. These regimes had the most cases and deaths per million, the highest fatality rate, the quickest outbreak response time, and the highest daily tests per thousand.

The WHO [20] stated that misinformation about COVID-19 posed a greater danger to public health than the virus itself. Arafat et al. [21] argued that false news on social media was a significant factor in causing panic buying. They suggested that bans from authorities could have prevented it. Additionally, Arafat et al. [22] studied media reports and found that 46% mentioned product shortages, 82% gave reasons for panic purchases, 80% spoke of the effects of such buying, and 26% reported rumours that led to it. They concluded that social media had a significant role in cases of panic buying.

Fear was the main factor driving the initial endorsement of restraining policies. Early in the pandemic, Bol et al. [23] found that lockdowns increased public support for governments, confidence in democracy, and approval of it in 15 European nations. Schraff [24] showed that higher COVID-19 metrics heightened Dutch political confidence due to anxiety. Marbach et al. [25] further found that lockdowns in the USA and four European nations increased support for authoritarianism and autocracy. Amat et al. [26] likewise found that Spanish survey data suggested public approval of a technocratic and totalitarian government style, even when it limited individual freedoms.

Existing research on COVID-19’s psychological impact may account for waning support for pandemic restrictions. Studies address domestic violence from lockdown-induced proximity, economic insecurity, and uncertainty [27]. Elevated anxiety, depression, and distress levels are also reported [28-30]. Adverse effects include posttraumatic stress, misperception, anger [31,32], and increased suicide rates [33-35]. Research has also examined COVID-19 patients’ feelings of guilt and shame [36].

The COVID-19 crisis disproportionately affected women, especially in the labour market. The World Economic Forum reported that USA women’s labour participation regressed to 1993 levels, with comparable declines in Japan and South Korea [37]. The Federal Reserve Board also identified significant increases in female workforce departures, predominantly among Black women, Latinas, and women with children [38]. Similarly, the UN (2020) informs that one of the negative impacts of the coronavirus crisis among women is the increased unpaid care workload due to children being out of school and the care needs of elderly family members. Likewise, the Office of Economic Cooperation and Development (OECD) [39] explains that the adverse effects of COVID-19 have been more severe among women, who represent about 70% of the health care workforce worldwide, exposing them to a higher risk of infection. The OECD also suggests that women have a more significant adverse pandemic impact due to school and childcare facility closures.

Equally, the International Monetary Fund also informs that women faced unbalanced effects from the coronavirus crisis due to its severe negative impact on economic sectors, with a significant share of women workers in the hospitality, retail, garment, and the food service industry [40]. Similarly, the World Bank found the pandemic’s labour market effects more harmful to women due to increased care work demands. Companies with top female managers faced disproportionately adverse impacts [41]. The International Labor Organization [42] reported a 4.2% global decrease in women’s employment from 2019-2020, compared to 3.0% for men, with the Americas and Arab countries experiencing the most significant declines.

## METHODS

The data for our independent variables were obtained from the World Bank’s [43] website. Our dependent variables include the female employment to population ratio, ages 15+ (Y1); the female employment to population ratio, ages 15-24 (Y2); and the female labour force as a percentage of the total labour force (Y3). The dependent variables also include the proportion of seats held by women in national parliaments (Y4); the share of youth not in education, employment, or training as a percentage of the female youth population (Y5); and the female unemployment as a percentage of the female labour force (Y6). We determined the annual change of these dependent variables as the difference between their values in the years 2019 (before the pandemic) and 2020 (the first year of the outbreak).

The difference between the dependent variables Y1 and Y2 is that the female employment to population ratio, ages +15, is a general measurement of most employed women. In contrast, the same ratio, but ages 15-24, intends to measure those mothers with infants, a population segment particularly vulnerable to the COVID-19 restrictions like schools’ and daycare centres’ closures. The dependent variable Y4 is a proxy measure of women’s political empowerment. The dependent variable Y6 is also a proxy measure of the percentage of women with unpaid care workloads.

Our independent variables include the total number of COVID-19 cases (X1) and deaths (X2) per million and the government’s annual average social restrictions index (X3). Similar to Erdem [44] and Herren et al. [45], we retrieved our independent and control variables’ data from the website Our World in Data, which is compiled by Mathieu et al. [46]. We determined the natural logarithm of the gross domestic product per capita (X4) as a control variable. We also retrieved global statistics from the Bloomberg database regarding corporate women’s empowerment from 2014 to 2021. Specifically, we obtained the yearly averages from all publicly traded companies (+90K) with available data in the Bloomberg database regarding the percentage of women on corporate boards, the percentage of women in executive and managerial positions, and the percentage of women employed in publicly traded companies.

We applied generalized linear models to analyse the cross-sectional variation of our dependent variables during 2020. Our models are determined by a linear predictor 𝜂𝑖 = 𝛽0 + 𝛽1𝐼𝑉𝐴𝑅1𝑖 + ⋯ + 𝛽𝑝𝐼𝑉𝐴𝑅𝑝𝑖; and two identities: a link equation that describes the mean (*Yi*) = *μi*, as a function of the linear predictor 𝑔(𝜇𝑖) = 𝜂𝑖; and a variance function that defines how the variance, var(Yi) relies on the mean var(Yi) = 𝜙𝑉(𝜇), where the distribution factor 𝜙 is a constant. For our general linear models, we have 𝜖 = (0,𝜎2), where the linear predictor 𝜂𝑖 has been defined above, the link equation 𝑔(𝜇𝑖) = 𝜇𝑖, and the variance equation 𝑉(𝜇𝑖) = 1. We also applied logarithmic transformations to some of our control variables when employing the regression models described above.

## RESULTS

**Figure 1:**
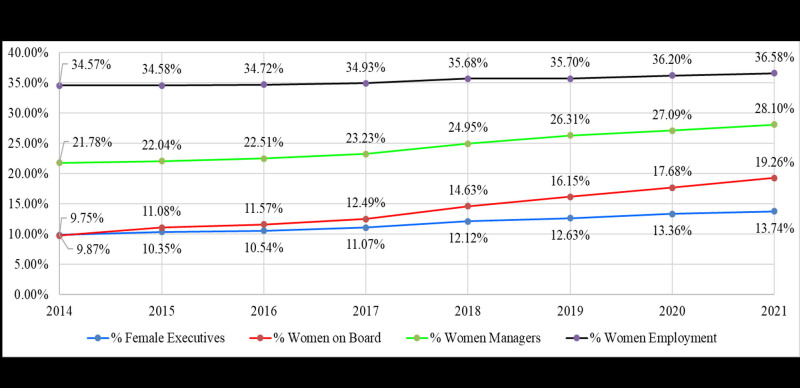
Women’s corporate empowerment over time. The lines above depict the global annual averages of women on corporate boards (red), the percentage of women in executive positions (blue), the percentage of women in managerial positions (green), and the percentage of women employed in publicly traded corporations (black).

**Figure 1** shows the global annual averages of women on corporate boards, the percentage of women in executive positions, the percentage of women in managerial positions, and the percentage of women employed in publicly traded corporations. The results suggest corporate women’s empowerment at all levels has increased yearly. In the specific case of COVID-19, all of these variables increased between 2019 and 2020. Each of these averages represents different corporate women’s empowerment levels among publicly traded companies. These averages reflect the recent tendency of corporate women’s empowerment at the board, executive, managerial, and employee levels. At each of these levels, corporate women’s empowerment shows a rising trend between 2014 and 2021, without a year where these averages were lower than the previous year. These results suggest that the pandemic had no adverse impact on corporate women’s empowerment from a global perspective.

**Figure 2:**
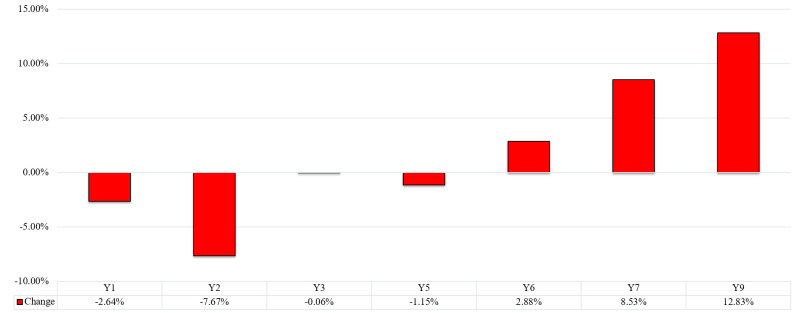
Change of dependent variables worldwide. Female employment to population ratio, ages 15+. Y2: female employment to population ratio, ages 15-24. Y3: Female labor force as a percentage of the total labour force. Y4: proportion of seats held by women in national parliaments. Y5: Share of youth not in education, employment, or training as a percentage of the female youth population. Y6: Female unemployment as a percentage of the female labour force.

**Figure 2** shows the percentage change of our dependent variables between 2019 and 2020 for our sample of 93 countries worldwide. The results show a decrease of 2.64% in the female employment to population ratio, ages 15+, and a reduction of 7.67% in the same female employment to population ratio but between ages 15-24. Similarly, the results show a small but statistically significant decrease of 0.06% in the female labour force as a percentage of the total labour force. Correspondingly, our results show an increase of 2.88% in the proportion of seats held by women in national parliaments. Likewise, the results show a rise of 8.53% in the share of youth not in education, employment, or training as a percentage of the female youth population and an increase of 12.83% in female unemployment as a percentage of the female labour force.

For analytical purposes, we ordered our sample by our dependent variables, from the lowest to the highest values, and identified the first and fourth quartiles. We then compared the averages of our dependent variables for each quartile. **Table 1** shows the independent samples test of our dependent variables for our sample of 93 countries. The statistically significant results show that countries with the greatest/lowest change in female employment to population ratio, ages +15, experienced a decrease / increase of 5.02% / 2.05% between 2019 and 2020. These same countries also endured the strictest/softest government social restrictions. Correspondingly, nations with the biggest / lowest change in female employment to population ratio, ages 15-24, also suffered a decrease / increase of 5.94% / -1.55% between 2019 and 2020. These same nations also endured the highest / lowest total COVID-19 cases and deaths per million.

**Table 1:** Independent samples test results*

Y1: Change of female employment to population ratio, ages 15+					
	**Y1**	**X1**	**X2**	**X3**	**X4**
Q1	-2.05	19 526.09	352.14	58.13	4.20
Q4	5.02	24 881.31	649.53	71.24	4.16
*t*-sta.	-8.48	-0.87	-1.94	-3.72	0.42
*P*-value	(0.0)†	(0.39)	(0.06)‖	(0.0)†	(0.68)
**Y2: Change of female employment to population ratio, ages 15-24**					
	**Y1**	**X1**	**X2**	**X3**	**X4**
Q1	-1.55	16 187.60	297.38	62.03	4.19
Q4	5.94	32 468.61	727.21	64.13	4.35
*t*-sta.	-7.90	-2.79	-2.95	-0.56	-1.48
*P*-val.	(0.0)†	(0.008)†	(0.005)†	(0.58)	(0.15)
**Y3: Change of female labour force as a percentage of the total labour force**					
	**Y1**	**X1**	**X2**	**X3**	**X4**
Q1	-0.15	20 176.67	329.98	55.96	4.17
Q4	0.85	17 385.01	362.95	67.75	3.90
*t*-sta.	-10.34	0.65	-0.34	-4.70	2.37
*P*-val.	(0.0)†	(0.52)	(0.73)	(0.0)†	(0.02)§
**Y4: Change in the proportion of seats held by women in national parliaments**					
	**Y1**	**X1**	**X2**	**X3**	**X4**
Q1	0.14	11 374.58	187.48	59.05	3.90
Q4	-1.27	21 350.34	472.11	57.40	4.11
*t*-sta.	2.57	-2.43	-3.15	0.53	-1.76
*P*-val.	(0.01)§	(0.02)§	(0.002)‡	(0.60)	(0.08)‖
**Y5: Change in the share of female youth, not in education, employment, or training as a percentage of the female youth population**					
	**Y1**	**X1**	**X2**	**X3**	**X4**
Q1	-0.50	34 468.58	563.21	52.68	4.63
Q4	-3.59	12 292.72	426.06	66.53	4.06
*t*-sta.	2.00	4.06	0.76	-4.60	7.17
*P*-val.	(0.05)§	(0.0)†	(0.45)	(0.0)†	(0.0)†
**Y6: Change in female unemployment as a percentage of the female labour force**					
	**Y1**	**X1**	**X2**	**X3**	**X4**
Q1	-0.05	18 056.42	321.82	57.18	4.36
Q4	-1.89	24 309.40	571.60	67.80	4.16
*t*-sta.	1.91	-1.12	-2.12	-2.99	1.90
*P*-val.	(0.06)‖	0.27	(0.04)§	(0.005)‡	(0.06)‖

*Our independent (X1-X3) & control (X4) variables are the total COVID-19 cases (X1) and deaths (X2) per million, the government’s annual average of social restrictions index (X3), and the natural logarithm of the gross domestic product per capita (X4). Q1 & Q4 denote the first and fourth quartiles, respectively.

†Statistical significance at 0.1% level.

‡Statistical significance at 1% level.

§Statistical significance at 5% level.

‖Statistical significance at 10% level.

The statistically significant results in **Table 1** also include countries with the biggest / lowest change in the female labour force as a percentage of the total labour force experienced a decrease / increase of 0.85% / 0.15% between 2019 and 2020. These countries had the highest/lowest natural logarithm of the gross domestic product per capita and tolerated the strictest / softest government social restrictions. Likewise, regimes with the greatest / lowest change in the proportion of seats held by women in national parliaments experienced a decrease / increase of 0.14% / 1.27% between 2019 and 2020. These same regimes also suffered the lowest/highest total COVID-19 cases and deaths per million. Equally, nations with the biggest / lowest change in the share of female youth not in education, employment, or training as a percentage of the female youth population experienced an increase of 3.69% / 0.50% between 2019 and 2020. These countries also had the lowest / highest natural logarithm of the gross domestic product per capita, endured the harshest / laxest government social restrictions, and suffered the lowest / highest total COVID-19 deaths per million. Finally, regimes with the greatest / lowest change in female unemployment as a percentage of the female labour force also experienced an increase of 1.89% / 0.05% between 2019 and 2020. These same regimes also suffered the highest / lowest total COVID-19 cases and deaths per million and the strictest / softest government social restrictions.

To avoid multicollinearity problems between total COVID-19 cases (X1) and deaths (X2) per million, we included these independent variables separately in our generalized linear models. **Table 2** and **Table 3** show the cross-sectional variation of our dependent variables (Y1-Y6) regressed against our independent (X1-X3) and control (X4) variables. The statistically significant results show a positive and significant relationship between the change in female employment to population ratio, ages +15, and the government social restrictions index, consistent with **Table 1**. The tables’ significant results also show a positive and significant relationship between the change in female employment to population ratio, ages 15-24, and total COVID-19 cases and deaths per million, consistent with **Table 1**. However, **Table 2** and **Table 3** also report this same positive and significant relationship between the change in female employment to population ratio, ages 15-24, and the natural logarithm of the gross domestic product per capita and the government social restrictions index. These last two relationships were insignificant in **Table 1**.

**Table 2:** Cross-sectional model including the total COVID-19 cases (X1) per million*

	Y1	Y2	Y3	Y4	Y5	Y6
C	-15.08	-38.65	0.48	-5.18	1.94	16.41
*z-sta.*	-2.08	-3.62	0.69	-1.14	0.13	1.96
*P-val.*	(0.04)§	(0.0)†	(0.49)	(0.25)	(0.89)	(0.05)§
X1	0.25	0.47	0.06	-0.03	0.45	-0.13
z-sta.	1.04	2.03	2.41	-0.21	1.49	-0.76
P-val.	(0.30)	(0.04)§	(0.02)§	(0.83)	(0.14)	(0.45)
X3	0.12	6.10	0.22	1.40	-0.47	-2.89
*z-sta.*	2.76	3.10	1.39	1.35	-0.18	-1.88
*P-val.*	(0.01)§	(0.002)‡	(0.16)	(0.18)	(0.86)	(0.05)§
X4	1.54	2.66	-0.38	-0.31	-1.31	-1.01
*z-sta.*	1.45	2.33	-4.17	-0.49	-0.81	-1.13
*P-val.*	(0.15)	(0.02)§	(0.0)†	(0.63)	(0.42)	(0.26)

*The dependent variables Y1-Y6 are the female employment to population ratio, ages 15+ (Y1); the female employment to population ratio, ages 15-24 (Y2); and the female labour force as a percentage of the total labour force (Y3). The dependent variables also include the proportion of seats held by women in national parliaments (Y4); the share of youth not in education, employment, or training as a percentage of the female youth population (Y5); and the female unemployment as a percentage of the female labour force (Y6). Our independent (X1 & X3) & control (X4) variables are the total COVID-19 cases (X1) per million, the government’s annual average of social restrictions index (X3), and the natural logarithm of the gross domestic product per capita (X4).

†Statistical significance at 0.1% level.

‡Statistical significance at 1% level.

§Statistical significance at 5% level.

**Table 3:** Cross-sectional model including the total COVID-19 deaths (X2) per million*

	Y1	Y2	Y3	Y4	Y5	Y6
C	-14.18	-32.45	0.65	-4.53	-0.80	17.20
*z-sta.*	-2.06	-2.99	0.88	-0.94	-0.05	1.96
*P-val.*	(0.04)§	(0.003)‡	(0.38)	(0.35)	(0.96)	(0.05)§
X2	0.37	0.41	0.07	0.01	0.20	-0.19
*z-sta.*	1.55	2.15	2.81	0.05	0.78	-1.24
*P-val.*	0.12	(0.03)§	(0.005)‡	(0.96)	(0.43)	(0.22)
X3	0.11	5.01	0.24	1.24	0.25	-3.16
*z-sta.*	2.73	2.50	1.46	1.16	0.09	-1.95
*P-val.*	(0.01)§	(0.01)§	(0.14)	(0.25)	(0.93)	(0.05)§
X4	1.52	2.80	-0.40	-0.37	-0.63	-0.99
*z-sta.*	1.48	2.61	-4.42	-0.63	-0.40	-1.14
*P-val.*	0.14	(0.009)‡	(0.0)†	(0.53)	(0.69)	(0.25)

*The dependent variables Y1-Y6 are the female employment to population ratio, ages 15+ (Y1); the female employment to population ratio, ages 15-24 (Y2); and the female labour force as a percentage of the total labour force (Y3). The dependent variables also include the proportion of seats held by women in national parliaments (Y4); the share of youth not in education, employment, or training as a percentage of the female youth population (Y5); and the female unemployment as a percentage of the female labour force (Y6). Our independent (X2 & X3) & control (X4) variables are the total COVID-19 deaths (X2) per million, the government’s annual average of social restrictions index (X3), and the natural logarithm of the gross domestic product per capita (X4).

†Statistical significance at 0.1% level.

‡Statistical significance at 1% level.

§Statistical significance at 5% level.

Similarly, **Table 2** and **Table 3** also show a positive and significant relationship between the change in the female labour force as a percentage of the total labour force and total COVID-19 cases and deaths per million, which is inconsistent with the results in **Table 1**, in which these relationships are insignificant. However, **Table 2** and **Table 3** also report a negative and significant relationship between the change in the female labour force as a percentage of the total labour and the natural logarithm of the gross domestic product per capita, consistent with the results in **Table 1**. Likewise, the tables’ significant results also show a negative and significant relationship between the change in female unemployment as a percentage of the female labour force and the government social restrictions index, consistent with the results in **Table 1**. Finally, **Table 2** and **Table 3** show no significant results between any of our independent variables and the change in the proportion of seats held by women in national parliaments and the change in the share of female youth, not in education, employment, or training as a percentage of the female youth population, which contradicts some results in **Table 1**.

## DISCUSSION

In conclusion, we studied the effects of the pandemic on women’s empowerment by analysing data from 93 countries worldwide between 2019 and 2020. We find a statistically significant decrease of 2.64% and 7.67% in the female employment to population ratio, ages 15+ and 15-24, respectively. Similarly, we find a small but statistically significant decrease of 0.06% in the female labour force as a percentage of the total labour force and an increase of 2.88% in the proportion of seats held by women in national parliaments. Likewise, we find a rise of 8.53% in the share of youth not in education, employment, or training as a percentage of the female youth population. Equally, we find an increase of 12.83 percent in female unemployment as a percentage of the female labour force. Correspondingly, we find strong statistical evidence of a positive and significant relationship between the change in female employment to population ratio, ages +15, and the government social restrictions index. Additionally, we find a positive and significant relationship between the change in female employment to population ratio, ages 15-24, and total COVID-19 cases and deaths per million. Finally, we verify a negative and significant relationship between the change in female unemployment as a percentage of the female labour force and the government social restrictions index.

Our results have substantial consequences at the community and government levels. Policymakers designing women empowerment strategies must validate their proposals based on scientific information. Women’s empowerment has become a globally relevant matter worldwide. Since the beginning of the 21st century, many countries have imposed gender quotas for corporate boards. Indeed, Kuzmina and Melentyeva [47] report national gender quotas for corporate boards in the UK (25% since 2015), France (40% since 2017), Italy (33% since 2015), Belgium (33% since 2017), the Netherlands (30% since 2016), Spain (40% since 2015), and Norway (40% since 2006). Likewise, Terjesen et al. [48] also inform on national gender quotas for corporate boards in Finland (40% since 2005), Quebec-Canada (50% since 2011), Israel (50% since 2010), Iceland (40% since 2013), and Kenya (33% since 2010). As recently as June 2022, the EU has decided to request publicly-traded companies with more than 250 employees to fill 40% of their directorship positions and 33% of their senior executive positions with women starting in 2026 [49]. Equally, in 2021 the US Securities and Exchange Commission authorized the National Association of Securities Automated Quotation System (NASDAQ) to ask firms listed in NASDAQ to include at least one woman on their board of directors. We examine all publicly traded companies (+90K) worldwide with available data in the Bloomberg database and find an increasing trend in the global annual percentage averages of women on corporate boards, executive positions, managerial positions, and women employed in publicly traded companies. Each of these averages represents different corporate women’s empowerment levels among public firms worldwide. Therefore, our results suggest that the impact of the COVID-19 crisis on corporate women’s empowerment was insignificant.

Alternatively, we find statistical evidence that the pandemic adversely affects several metrics of women’s empowerment at the community level. Our results about a decrease of 2.64% in the female employment to population ratio, ages 15+, and a decrease of 0.06% in the female labour force as a percentage of the total labour force both represent valid scientific evidence for national and local governments to support and protect women’s employment as a fundamental enabling factor for women’s empowerment. Our solid statistical evidence of a positive and significant relationship between the change in female employment to population ratio, ages 15-24, and total COVID-19 cases and deaths per million suggest that this specific population segment was particularly vulnerable in countries severely impacted by the coronavirus crisis with restrictions like schools’ and daycare centres’ closures. Similarly, our findings about an increase of 8.53% in the share of youth not in education, employment, or training as a percentage of the female youth population suggest that the pandemic may have increased the women’s unpaid care workload because of children out of school and the care needs of elderly family members as indicated by the UN [50]. These statistical results can justify policies to increase government support for family services in times of crisis.

Lastly, our results about an increase of 2.88% in the proportion of seats held by women in national parliaments support the anecdotal evidence regarding the public perception of enhanced women political leaders’ performance at controlling COVID-19. Indeed, in an article titled Are female leaders more successful at managing the coronavirus crisis? in The Guardian, Henley and Roy [51] report that women-led nations exhibited the most outstanding performance at managing the pandemic. These countries included Silveria Jacobs (Prime Minister of Saint Maarten), Jacinda Ardern (Prime Minister of New Zealand), Angela Merkel (Chancellor of Germany), Mette Frederiksen (Prime Minister of Denmark), Tsai Ing-wen (Taiwan’s president), Erna Solberg (Prime Minister of Norway), and Sanna Marin (Prime Minister of Finland).

One limitation of our study was the inclusion of the female employment-population ratio for ages 15-24, driven by the accessibility of this secondary data within the World Bank’s database for all nations and years examined. Ideally, the age range should have been 15-35; however, given the constraints imposed by available secondary data sources, it is prudent to exercise caution when generalizing that the 15-24 age group is the most suitable representation of mothers with infants. Further investigation is warranted to bolster confidence in the assertion that the population of mothers with infants was especially susceptible to the impacts of COVID-19 restrictions, such as school and daycare centre closures. Similarly, Morris and Reuben [52] provide some restrictions concerning efforts for international pandemic comparison. They report irregularities in COVID-19 countries’ cases and death records, variances in national testing programs, discrepancies in public health systems, unreliable data from some less democratic countries, and numerous demographic factors influencing the outbreak like age mean profile, population density, urban vs rural residents, age pyramid, etcetera.
